# Assessment of Bone Loss Around Platform-Switching Subcrestal Implants: A Quasi-Experimental Study

**DOI:** 10.7759/cureus.71460

**Published:** 2024-10-14

**Authors:** Divya Yadav, Rohit Goyal, Lakhan Talreja, Gollamudi Ramya, Sangita Kalita, Amit Kale

**Affiliations:** 1 Department of Oral and Maxillofacial Surgery, Maharaja Ganga Singh Dental College and Research Centre, Sriganganagar, IND; 2 Department of Conservative Dentistry and Endodontics, Maharaja Ganga Singh Dental College and Research Centre, Sriganganagar, IND; 3 Department of Prosthodontics, Maharaja Ganga Singh Dental College and Research Centre, Sriganganagar, IND

**Keywords:** bone loss, brushing, dental implants, follow up, periodontal index, stability

## Abstract

Introduction: Replacement of lost teeth with implants is a well-known and accepted worldwide treatment. A healthy amount of bone surrounding the implant plays a vital role in osseointegration and is required for implant success. This study aimed to evaluate the efficacy of single-crown rehabilitation of subcrestal implants in terms of bone loss (BL) and overall success.

Materials and methods: Twenty healthy patients requiring implant placement to replace single hopeless teeth or extracted teeth were recruited for this prospective study. In these 20 patients, the implants were placed at the subcrestal level. Bone levels around the implant placement (T0) and one year after loading (T1) were estimated in this study. A paired t-test was used for intra-group comparisons, multivariate linear regression analysis was conducted to analyze the effect of independent variables on BL, and a correlation test was used to correlate various variables. Statistical significance was maintained at a p value of 0.05.

Results: The outcomes demonstrated statistically significant BL at the mesial and distal sides of the subcrestal implants at one-year follow-up (p<0.05). Age and sex were not significantly correlated with BL in any region (p>0.05). Brushing frequency, probing depth (PD), and bleeding index (BI) showed statistically significant effects on BL (p<0.05). A weak correlation was observed between age and other variables, with age and mesial BL showing a low correlation of 0.016.

Conclusion: Within the parameters of this prospective study, it could be proposed that subcrestal implants caused significant BL. The PD, BI, and brushing frequency were significant predictors of BL.

## Introduction

Dental implant therapy has been established as a dependable approach for restoring edentulous spaces, primarily due to osseointegration, a vital biological and biophysical mechanism. The primary aim of implant placement is to establish and sustain a close interface between the bone and implant, a process referred to as dental osseointegration [1]. Clinically, dental osseointegration is defined as asymptomatic and rigid fixation of the implant within the bone, possessing sufficient strength to withstand masticatory forces. For osseointegration to transpire effectively, it is imperative to have an adequate volume of viable bone around the implant to facilitate the healing and integration process. Optimal osseointegration necessitates meticulous planning, exacting surgical methodologies, and consideration of various biological determinants [2].

The strategic placement of dental implants, especially concerning their relationship to the alveolar bone crest, has been extensively examined and debated in academic literature. Implants may be positioned at varying heights relative to the bone crest, encompassing supracrestal, crestal, and subcrestal placements [3]. Each of these positioning methodologies presents distinct advantages and disadvantages, particularly regarding the risk of peri-implant bone loss (BL), which is a crucial factor for the long-term efficacy of the implant. Peri-implant BL denotes the resorption of osseous tissue surrounding the implant and is affected by multiple elements, including the design of the implant, technique employed during surgery, biomechanical stress applied, and depth of implant insertion [4].

Recently, an increasing number of studies have focused on subcrestal implant placement (SIP), wherein the implant is strategically positioned slightly beneath the level of the bone crest. This technique has been advocated as a method to enhance aesthetic results by improving soft tissue coverage and facilitating a more natural emergence profile of the prosthetic crown [3]. SIP is posited as a tactical approach to minimize the exposure of the rough surface, as implants situated sub-crestally are more likely to maintain their position over extended durations [5]. Numerous investigations have demonstrated that non-submerged implant systems are correlated with reduced BL compared to submerged systems, which is attributed to the absence of a microgap at or beneath the alveolar crest [5,6]. Nevertheless, apprehensions have been expressed regarding the possibility of heightened BL associated with SIP [7]. Consequently, the primary aim of the current investigation was to evaluate BL and the comprehensive success of SIP one year after loading (T1). Through a comparative analysis of peri-implant bone levels at the moment of implant insertion and one year after the loading phase, this study elucidates the long-term clinical efficacy of SIP in terms of bone conservation and overall success.

## Materials and methods

Study design and setting

This quasi-experimental study was conducted in the Department of Oral and Maxillofacial Surgery at Maharaja Ganga Singh Dental College and Research Centre, Sriganganagar, Rajasthan, from April 2022 to November 2023. Institutional ethics committee approval (MGSDC/SY/22/018) was obtained before starting the study, and the study was conducted in accordance with the principles of the Declaration of Helsinki. Written consent was obtained from all participants prior to treatment.

Sample size estimation

The requisite sample size was determined using G*Power statistical software (Ver. 3.6.9 Heinrich-Heine-Universität Düsseldorf, Düsseldorf, Germany) utilizing an effect size of 0.57 from a prior study showing a mean BL of 0.145 mm in one year with a combined standard deviation (SD) of 0.25 [8]. To achieve an 80% statistical power (β=0.20) and a 5% significance level (α error), a two-tailed analysis indicated that a sample of 20 participants is sufficient to identify a statistically significant difference between interval periods.

Patient selection and group allocation

Twenty healthy adult patients participated in this study. The inclusion criteria required patients aged ≥18 years to have at least a single missing tooth between two adjacent natural teeth (extracted at least six months before placing the implants) that required replacement with a dental implant, presence of Angle’s class I occlusion with no parafunctional habits, opposing natural teeth, bone quality of D2 and D3 as determined by an operator during the time of planning for implant placement, bleeding index (BI) <1, and probing depth (PD) <3 mm [9-11]. Excluded from the study were patients with the following: systemic conditions affecting bone healing, a history of bone disorders and alcohol consumption, multiple missing teeth, temporomandibular joint disorders, and orofacial abnormalities. Patients undergoing bisphosphonate therapy, taking corticosteroids, using antibiotics in the past six months, and undergoing radiation therapy were excluded. Also excluded were smokers, edentulous patients, pregnant and lactating females, and those who needed guided bone regeneration around dental implants. The teeth were extracted due to endodontic problems.

Implant placement procedure

All patients underwent implant placement performed by an experienced oral surgeon under local anesthesia. A standardized surgical protocol was followed for each patient. The implants were placed at subcrestal level approximately 1 to 2 mm below the alveolar bone crest in the edentulous region of the jaw. The implants used in this study were of the same brand and model: Touareg™-S (Adin Dental Implant Systems Ltd., Afula, Israel) of diameter 4.5 or 5.0 mm and length 8.5 or 10 mm (depending on bone availability) made of titanium with a roughened surface, featuring a neck design with microthreads, a treated surface, internal connection, and platform switching that has been shown to enhance osseointegration. In platform switching design, the narrower abutment is positioned atop a broader implant platform, typically exhibiting a discrepancy of approximately 0.5 to 1 mm between the diameters of the implant and the abutment.

A two-stage surgical approach was employed, where the surgical site was closed with sutures after implant insertion. A healing period of three months was allowed for osseointegration to occur. Postsurgical care instructions were provided to all patients, including antibiotic therapy, pain management, and oral hygiene practices.

Prosthetic rehabilitation

After the osseointegration period, second-stage surgery was performed to expose the implants, and healing abutments were placed. Following soft tissue healing, a single-crown prosthesis was fabricated and delivered to each patient. According to the manufacturer's guidelines, all screws were tightened to a torque of 30 cm. The crowns were cement-retained and designed to provide optimal function and esthetics. After the final crown placement, implants were loaded, and patients were instructed on proper oral hygiene techniques and scheduled for regular follow-up visits.

Method of clinical and radiographical evaluation

**Figure 1:**
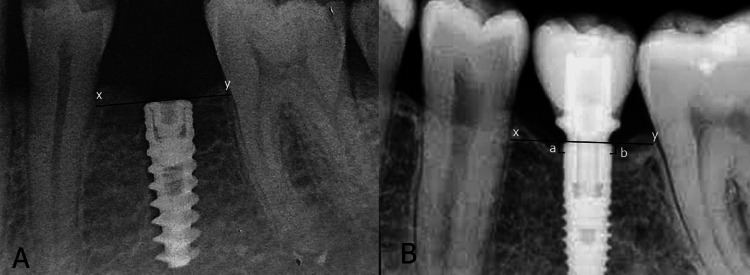
Level of marginal bone (A) at T0 and (B) at T1 where XY line is a line at implant platform The image is of a patient involved in the study. T0: Time of implant placement; T1: One-year follow-up after loading

All patient information, such as sex, age at implant placement, implant length, implant diameter, and frequency of brushing, was archived through a normative process. The radiographs were elucidated at the time of implant placement (T0) and T1 (Figure 1).

**Figure 2:**
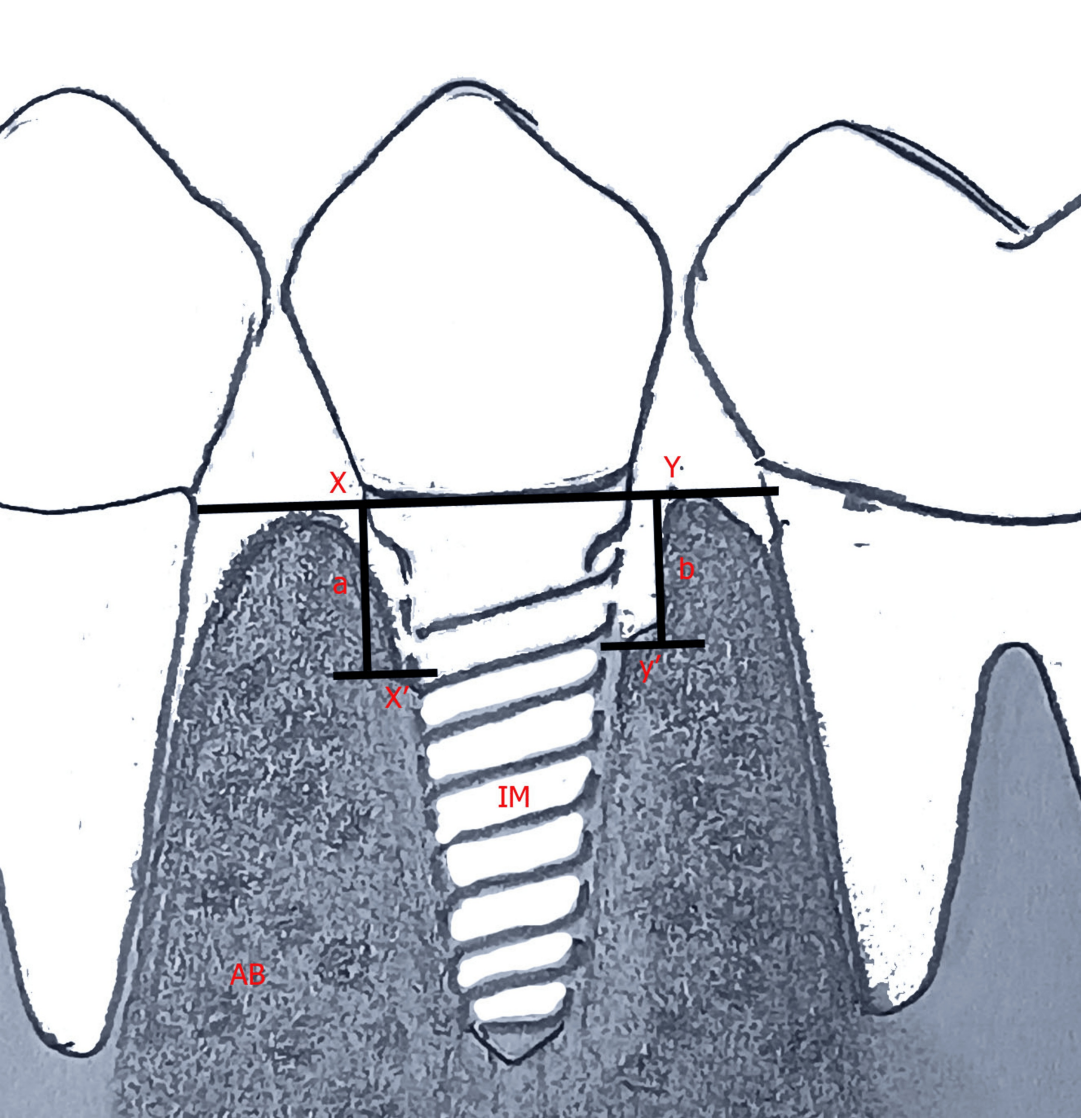
Assessment of marginal BL on mesial and distal sides of subcrestal implants The author created the original image. BL: Bone loss; IM: Implant, AB: Alveolar bone; XY line: Line at implant platform; x': Point of contact of alveolar bone on mesial side; y': Point of contact of alveolar bone on distal side; a: BL on mesial side (mm); b: BL on distal side (mm)

The radiographic evaluation was conducted employing a RadioVisioGraphy (RVG) intraoral digital receptor (Vatech Co. Ltd., Gyeonggi-do, South Korea). To replicate patient configurations, the Rinn XCP-DS Fit® positioning system (Dentsply, Des Plaines, IL, US) consisting of rigid bars, bite blocks, and rings for proper alignment was used. The receptor was retained within a slot incorporated in the bar. Two easily visible and regional reference locations were selected at the implant platform for measurement. Taking two reference points, a straight line was marked and considered zero height. To calculate the amount of BL, a perpendicular line was drawn from zero height to the point of contact with the bone, both mesially and distally (Figure 2).

To evaluate the BL on the mesial and distal sides during implantation, the mean difference of values between T1 and T0 was calculated and tabulated for statistical analysis. Implant success was defined based on clinical and radiography-based standards described by Buser et al. [12].

Statistical analysis

Data analysis was conducted using IBM Statistical Package for Social Sciences (SPSS) Statistics software for Windows version 22.0 released in 2013 (IBM Corp., Armonk, NY, US). Continuous variables are reported as mean and SD. The normality of the data was assessed using the Shapiro-Wilk test, which confirmed that the data followed a normal distribution. As the data were continuous and normally distributed, parametric tests were employed for inferential statistics. A paired t-test was used to examine BL during a year interval within the group. Multivariate linear regression analysis was conducted to analyze the effect of independent variables on BL. Pearson correlation test was used to correlate the variables, such as age, BI, and PD. The Biserial correlation test was used for brushing frequency and diet.

## Results

The descriptive analysis of the study sample revealed that the sample consisted of 10 (50%) males and 10 (50%) females with a mean age of 37.60 ± 7.82 years. 12 (60%) were vegetarians. Brushing was performed once daily in 13 (65%) patients. The implant side was mostly on the right side in 12 (60%) patients, and all were first molars, mainly in the mandibular jaw. The most commonly used implants were 10 mm and diameter 4.5 mm (Table 1).

**Table 1:** Descriptive analysis of independent variables. Data is presented in the form of n (%).

Variables	Category	Frequency (n)	Percentage (%)
Sex	Male	10	50%
	Female	10	50%
Diet	Veg	12	60%
	Non-veg	8	40%
Implant side	Right	12	60%
	Left	8	40%
Jaw	Maxillary	7	35%
	Mandibular	13	65%
Brushing frequency	Once	13	65%
	Twice	7	35%
Implant length (mm)	8.5 mm	4	17%
	10 mm	20	83%
Implant diameter (mm)	4.5 mm	13	54%
	5 mm	11	46%

A total of 100% of the implants were successful during the follow-up period. The comparison of BL for SIP during the year follow-up was analyzed using a paired t-test. Significant BL was observed on both the mesial and distal sides (p=0.001). Cohen's d values indicated a large effect size. More BL were observed on the distal side than on the mesial side. This showed that at one-year post-prosthesis follow-up, BL was significantly present around the dental implants (Table 2).

**Table 2:** Comparison of BL in SIP at T0 and T1 by paired t-test. Data presented in form of mean ± SD. p value ≤ 0.05: Significant BL: Bone loss; BI: Bleeding index; PD: Probing depth; MD: Mean difference; SIP: Subcrestal implant placement; T0: Time of implant placement as baseline; T1: One year after loading; SD: Standard deviation

Parameters	T1-T0 (MD)	p Value	Effect Size
Mesial BL (mm)	1.17 ± 0.157	0.001*	1.35
Distal BL (mm)	1.21±0.016	0.001*	1.1
BI	1.58±0.64	0.023*	0.28
PD (mm)	2.05±0.58	0.028*	1.36

Multivariate linear regression analysis was used to examine the influence of various factors on the BL. Age and sex were not significantly correlated with BL in any region (p>0.05). Brushing frequency, PD, and BI showed statistically significant effects on BL on both the mesial and distal sides (p<0.05). Overall results highlighted specific correlations between BL and factors such as brushing frequency, BI, and PD (Table 3).

**Table 3:** Multivariate linear regression analysis p value ≤ 0.05: Significant BL: Bone loss; PD: Probing depth; BI: Bleeding index

Variables	Mesial BL		Distal BL	
Coefficient with p value	Coefficient	p value	Coefficient	p value
Age	-00205	0.878	-0.00153	0.079
Sex	1.134	0.90	7.78	0.550
Brushing frequency	0.03988	0.018*	0.0799	0.03*
Diet	10.18	0.363	6.51	0.649
PD	0.00076	0.018*	0.01369	0.002*
BI	-0.000003	0.024*	-0.00859	0.032*

The correlation test revealed correlations between the various clinical parameters. (p<0.05). PD showed a positive correlation with the mesial and distal BL (p<0.05). BI showed a positive correlation with mesial BL (p<0.05), while other factors did not show any statistically significant correlation with any other factor (p>0.05) (Table 4).

**Table 4:** Correlation analysis between independent and dependent variables p value ≤ 0.05: Significant BL: Bone loss; PD: Probing depth; BI: Bleeding index

Variables	Correlation Test	Mesial BL	Distal BL
Age	Pearson's r	0.12	0.24
	p value	0.632	0.746
Brushing frequency	Biserial	0.02	0.14
	p value	0.146	0.328
Diet	Biserial	0.23	0.05
	p value	0.627	0.721
BI	Pearson's r	0.23	0.023
	p value	0.043*	0.068
PD	Pearson's r	0.45	0.23
	p value	0.027*	0.041*

## Discussion

The present study provides valuable insights into the BL surrounding subcrestal implants, analyzed over a one-year follow-up period. Understanding the patterns of BL around implants and the factors influencing it is crucial for improving clinical outcomes. This study aimed to examine the BL in patients with single-implant prostheses.

The study observed significant BL on the mesial and distal sides of the implants during T1. The results, analyzed using a paired t-test, revealed a statistically significant difference in the bone levels at different time points (p=0.001). It indicates that bone resorption around dental implants was substantial during T1 and prosthesis loading. Kütan et al. reported 1.22 mm of BL at a three-year follow-up period [6]. Similarly, Pellicer-Chover et al. reported a 1.22 mm BL after 12 months of follow-up [7]. A 100% success rate was noticed in this study, in accordance with previous studies [12,13].

The SIP in this study was performed with implants with a roughneck design and platform switching associated with less BL than the standard design [14]. The reason for the significant BL observed in this study might be that when implants are positioned subcrestally, they are situated beneath the crest of the alveolar bone, necessitating bone remodeling to accommodate the implant's location. This bone remodeling may result in initial BL. This phenomenon is particularly evident in subcrestal implants, where the peri-implant bone is required to adapt to the deeper positioning of the implant platform, leading to more bone resorption during the healing process. The deeper insertion of the implant into the bone results in the abutment and prosthesis being positioned further away from the bone, potentially generating a longer lever arm that exerts increased stress on the bone-implant interface. In this study, most were molar implants. Therefore, this heightened stress can exacerbate BL, especially when the implant endures functional occlusal loading, notably in regions subjected to intense chewing forces. However, in the case of subcrestal implants, the efficacy of platform switching may be compromised. The deeper positioning of the implant implies that the inflammatory response may occur closer to the bone, lessening the protective benefits of platform switching and consequently leading to BL [15].

A contemporary systematic review asserted the importance of conical connections in sealing efficiency, micro gap formation, torque preservation, and stability of the prosthetic abutment. These observations indicate that macro- and micro-design elements of the implant may significantly influence alterations in the marginal peri-implant bone when implants are positioned subcrestally [16]. A noteworthy finding of this study was the difference in the BL between the mesial and distal sides of the implants. The distal side exhibited a more pronounced BL than the mesial side. This discrepancy may be attributed to several biomechanical factors. The distal side of an implant often endures more occlusal force because of its anatomical position in the dental arch and proximity to the second molar, which may contribute to accelerated bone resorption. Additionally, the implant-abutment connection and micromovements during functional loading may be more pronounced on the distal side, leading to more BL in this region. These findings are consistent with previous studies that reported similar patterns of asymmetric BL around implants, with the distal side being more vulnerable to occlusal overload and peri-implantitis [17,18].

Increased PD and BI were observed, which were significant predictors of BL in this study. Brushing frequency was also a significant predictor and most of the patients brushed once daily, which could be a reason for the increased PD and BI scores. PD is an indicator of periodontal health, and its increase suggests the progression of peri-implant inflammation, which in turn exacerbates bone resorption [19]. The significant relationship between PD and BL highlights the importance of regular periodontal assessment in patients with dental implants to detect the early signs of peri-implant disease. Early intervention can prevent further BL and ensure long-term implant stability.

Clinical implications

This study highlights the importance of patient education in maintaining good oral hygiene practices, such as regular brushing to prevent peri-implantitis and BL. Additionally, it underscores the need for regular periodontal assessments, particularly monitoring of PD and BI, to detect early signs of inflammation that could lead to BL. Clinicians should also pay close attention to the distal side of the implant, which appears more susceptible to BL than the mesial side, possibly because of occlusal factors.

Limitations

Although the results of this study provide valuable insights, there are some limitations to consider. The sample size was relatively small, which may limit the generalizability of our findings to larger populations. Additionally, the study only followed patients for one year after implant loading. Longer follow-up periods would provide a more comprehensive understanding of the long-term patterns of BL. Finally, the study did not include a detailed occlusal analysis, which could have further explained the differences in mesial and distal BL.

## Conclusions

In summary, this present study revealed significant BL on the mesial and distal sides with SIP over a one-year monitoring duration, with the distal aspect exhibiting more significant bone resorption. Clinical parameters, such as frequency of brushing, PD, and BI, were identified as significant predictors of BL, whereas age and sex appeared to have no effect. This highlights the importance of maintaining proper oral hygiene and regular follow-up for SIP success.
